# Misconduct in science and medicine

**DOI:** 10.1530/EOR-2025-0126

**Published:** 2025-07-01

**Authors:** Alain Charles Masquelet

**Affiliations:** National Academy of Medicine, Paris, France

**Keywords:** orthopedics, orthopaedics, misconduct, Covid, Covid-19

One positive aspect of the Covid-19 health crisis has been to highlight for us all the fact that science – and in particular medical science – is not a long, placid river, both majestic and pristine. Within the first few months of the pandemic, the Covid health crisis brought to light a problem that is, if not taboo, at least in the main concealed. The problem answers to many names: imposture, mystification, deception. But by any name, it is, and remains, what it is: fraud…scientific fraud**.**

Let us go back to early 2020, when France imposed its first lockdown. That lockdown lasted 3 months, and in June, the country was thrown into turmoil as uninformed readers of the mainstream press discovered with astonishment that medical science could be fraught not only with errors of all types – of course – but also misconduct and even misdeeds.

It all started with a warning – an ‘expression of concern’ (1) – published in *The Lancet* dated June third, with regard to an article the journal had published less than 2 weeks prior (2). Now, any article that appears in *The Lancet* is, *a priori*, trustworthy. At issue was an article, published May 22, that reported on a study based on the electronic medical records of 96,000 patients who had been hospitalised with Covid-19 (3). The study strongly suggested that patients treated with chloroquine or hydroxychloroquine had both a higher mortality rate and more cardiac troubles; and it was this article that prompted the World Health Organization to suspend the inclusion of patients treated with chloroquine in its international Solidarity clinical trial. Similarly, in France, Minister Olivier Véran seized the High Council of Public Health. The council followed suit by declaring itself unfavourable to the use of chloroquine, at least in a hospital context – in like manner, the Discovery clinical trial launched by INSERM was also suspended.

A certain Mandeep Mehra of Harvard Medical School had conducted the study in question. It claimed to be based on data from a company named Surgisphere, founded by a surgeon named Sapan Desai.

Desai claimed to have collected 96,000 medical records from 671 hospitals on six continents – including Africa, where computer equipment is far from the rule. One hundred and twenty researchers signed an open letter demanding access to the raw data, in order to reanalyse them and even verify their authenticity. The leaders of Surgisphere, citing legal reasons, deemed the request inadmissible. In France, Didier Raoult described the study as ‘half-baked’ and said its authors had ‘shot themselves in the foot'.

This was not Sepan Desai’s first attempt to see his company’s data serve as the keystone for a scientific paper’s conclusions. He had co-authored an article published in the *New England Journal of Medicine* on May 1 (4). With results based on data provided by Surgisphere, gleaned from the electronic files of 169 hospitals on three continents, the study concluded that taking antihypertensive drugs beforehand had no effect on mortality from Covid-19. The *New England Journal of Medicine*, prefiguring *The Lancet’s* expression of concern, soon issued an alert to readers, citing ‘significant concerns raised about the quality of information contained in the database’.

To make a long story short, *The Lancet’s* warning turned into a genuine Lancet-gate, exacerbated by the social networks. On June 4, 2020, *The Lancet’s* editorial team, led by Richard Horton, decided to retract the article, just two days after it had been published.

Retract…This was not the first time *The Lancet* had retracted an article originally published in its columns. In 1998, the famous journal published a study based on the cases of twelve children, claiming that some had developed a form of autism following the administration of the measles–mumps–rubella vaccine (5). The article’s publication prompted a sharp decline in vaccination rates in England and a subsequent increase in measles cases. In reality, a lawyer wishing to take action against a laboratory had bought the author, Andrew Wakefield. In 2004, The Sunday Times revealed that the autistic children had already been diagnosed as having autism before being vaccinated. In 2010, a British Medical Council court found the doctor guilty of fabricating data and withdrew his right to practise medicine in the United Kingdom for life. Wakefield, who has always denied the fraud, now practises in the United States, where he regularly intervenes for the benefit of anti-vaccine lobbies.

In 2006, *The Lancet* was again seen to retract an article, after its author, a university dentist from Oslo named Jon Sudbø, admitted to having invented data for an analysis concluding that non-steroidal anti-inflammatory drugs reduced the risk of oral cancer (6). As editor Richard Horton commented at the time, ‘The peer-review process is good at picking up poorly designed studies, but it is not designed to pick up fabricated research. Just as in society you cannot always prevent crime; in science you cannot always prevent fabrication’.

Hence, there is no end to such fraud – and it affects all scientific disciplines.

Cyril Burt, a renowned British psychologist, devoted much of his career to proving that intelligence was hereditary by comparing the IQ test scores of identical twins. The publication of his study strongly influenced public education policy in the United Kingdom. It was later discovered that some of those twins never existed (7).

In an article published in *Nature* in 2001, Jan Hendrik Schön, a young German physicist, claimed that he had succeeded in developing a molecular transistor (8). Several teams were unable to replicate the results, and after pleading error in good faith, Schön was forced to admit that he had tweaked the results to make them more convincing.

In 2006, a Korean researcher, Hwang Woo-Suk, known to be an expert in cloning, made scientific headlines (9). Hwang claimed that he had succeeded in cloning human cells, a breakthrough that would have opened up promising prospects for research. Various laboratories tried in vain to reproduce his results, until an American team came to the conclusion that without realising it, the Korean researcher had obtained human embryonic cells not by cloning, as he claimed, but by parthenogenesis. The Korean protocol used for the research made the distinction difficult, but Hwang was criticised for not carrying out basic checks.

In much the same way, the Italian-born cardiologist Piero Anversa of Harvard Medical School claimed in a resounding article published by the* New England Journal of Medicine* that myocytes, the cells of the heart muscle, were capable of regeneration (10). Since no other team was able to replicate the Harvard team’s results, Anversa’s papers were retracted due to falsified and/or fabricated data, and the majority of the team’s collaborators left Harvard after shutting down the lab.

But the world record for scientific cheating is held by Japanese anaesthesiology researcher Yoshitaka Fujii, who was expelled from the University of Tokyo in 2012 for having doctored no fewer than 180 papers on the alleged preventive effect of granisetron on postoperative nausea and vomiting (11).

What about my homeland, the celebrated Patrie des Lumières?

Is France immune to cases of fraud?

Let us consider a few emblematic cases.

Serge Voronoff – a surgeon of Russian origin, naturalised French, who had done an internship in New York with Alexis Carrel – was certainly a pioneer in the technique of grafts, in particular bone grafts, which he performed during World WarI. Between the two world wars, a large portion of his activity was devoted to grafting tissue taken from monkey testicles into men of a certain age to restore their youth, strength, and vigour (12). During the 1930s, more than 500 men were treated in France with this rejuvenation technique, including numerous personalities and politicians. Although many patients were grateful (probably due to the placebo effect), it turned out that none of the xenograft operations produced the expected results.

From the more recent past, some will remember the Benveniste affair, an immunologist convinced that water possessed a molecular memory – which, had it been real, would have ensured the happiness and fortune of homoeopaths. Unfortunately, none of Benveniste’s experimental results could be replicated (13). Despite this experimental refutation, the water memory theory still has many followers – including the illustrious and late Luc Montagnier, who gave credence to the theory in the twilight of his life, long after he received the Nobel Prize in Medicine for his discovery of the AIDS virus (14). It goes to show that even the greatest minds can go astray.

Even more recently, biologist Anne Peyroche, appointed interim president of the CNRS in October 2017, was implicated soon afterwards by the PubPeer website for manipulation of data (15). This eventually earned her a 2-week suspension.

In this long list, it is impossible not to mention Didier Raoult, a fierce supporter of treating Covid-19 patients with a combination of hydroxychloroquine and a macrolide. Inspired by several protocols that had been tried in China before the pandemic reached Europe, Raoult forged his intimate conviction of hydroxychloroquine’s efficacy on a short retrospective series (16). However, with a disease such as Covid-19, where the lethality rate is rather low, only randomised trials can demonstrate the effectiveness of a given treatment. The IHU Méditerranée Infection in Marseille had not produced solid data. Moreover, Didier Raoult ignored the negative opinion delivered by a Committee for the Protection of Persons.

In like manner, l’Assistance Publique – Hôpitaux de Paris was hasty in announcing promising results for tocilizumab in the treatment of severe forms of Covid (https://www.aphp.fr/contenu/le-tocilizumab-ameliore-significativement-le-pronostic-des-patients-avec-pneumonie-covid). An independent committee responsible for monitoring the trial had found inconsistencies in the data. They felt that the trial should be continued before deciding. Ignoring the committee’s recommendation, l’Assistance Publique – Hôpitaux de Paris made their premature announcement anyway.

Finally, at the risk of denting an iconic French scientist’s halo, we must mention the case involving Pasteur. The year: 1885. The date: July 6, when Louis Pasteur administered the first ever inoculation against rabies to a young Alsatian boy named Joseph Meister, badly bitten on the arms and legs by a dog that was subsequently shot dead. The basis of Pasteur’s vaccine is an attenuation of rabies contained in rabbit spinal cord infected with the virus. Previously, Pasteur had carried out an experiment on some two dozen dogs, with cures in approximately 75% of the cases. By current standards, such results are not sufficiently significant. Indeed, his faithful collaborator Dr Roux dissociated himself from Pasteur for this first human trial, because if the boy was not infected, he ran a high risk of catching the disease from the inoculation itself. Notably, only 16% of patients who are bitten by a rabid dog actually contract the disease. Moreover, it was by no means certain that the dog that bit young Meister was well and truly rabid… and we will never know, because the brain bulb of the dog was not taken for inoculation into rabbits. Nevertheless, Joseph Meister survived, and the event ensured Pasteur’s mythical fame.

But in this incredible history of rabies vaccination, there is another even more notable episode. On October 8, 1886, an unknown dog bites a 12-year-old boy named Jules Rouyer. The child is brought to Pasteur, who performs an inoculation. Seven weeks later, on November 26, the child dies. So, the question arises: should the boy’s death be attributed to the bite or to Pasteur’s inoculation? Forensic expertise is entrusted to Paul Brouardel, who holds the chair of forensic medicine in Paris. He is close to Pasteur and has been an early proponent of the chemist’s theses. The sample taken from the bulb of the child is inoculated into two rabbits. Both rabbits die. There can be no doubt. The boy had rabies: either the inoculation failed or it was the cause of the infection. In January 1887, during a meeting of the Académie de Médecine, Brouardel reads the report he has addressed to the public prosecutor. In the report, he affirms that the two rabbits inoculated with the bulb of the child are in good health 42 days after the inoculation. On the basis of that report, the hypothesis that the child had rabies is ruled out, and his death is attributed to a crisis of uraemia. Some time prior, Brouardel had confided to Pasteur and his collaborators: ‘If I do not take a position in your favour, it is an immediate setback of 50 years in the evolution of science. This must be avoided’. Thus, pragmatic considerations – the Institute must not be harmed, Pasteur’s reputation must not be tarnished – prevailed over scientific rigour and honesty. The fact of the matter is that Brouardel perjured himself. His report was fraudulent. The conclusion I personally draw is that Pasteur, who was a chemist and not a doctor, was never careful enough when it came to medical matters. It is reasonable to think that if he had been a doctor, he would have taken additional precautions in view of possible failures (17, 18).

However, all of these more or less serious misconducts in terms of scientific rigour that we have so far enumerated can hardly compare to the perversion of science by ideology, and there is no better example of this than Lyssenkism. Named after a Soviet agricultural specialist who was favoured by Stalin in the 1930s, Lyssenkism was a veritable mystification. Under the pretext that genetics was an offshoot of Western capitalism, and by manipulating different kinds of wheat, Trofim Lyssenko claimed to show that genes did not exist. In Lyssenko’s view, geneticists were the enemies of the people and should be sent to jail, if not liquidated. A golden opportunity to get rid of potential rivals (19)!

These stories are hardly anecdotal. Indeed, they testify to a phenomenon which has always existed, as demonstrated by three fraudsters famous for their discoveries and who have gone down in history.

To begin with, Ptolemy, reputedly the greatest astronomer of antiquity, who described a geocentric system of the movement of the stars according to measurements which he claimed to have carried out himself – whereas they had, in fact, been carried out 300 years earlier by the Greek astronomer Hipparchus on the island of Rhodes (20). This is called plagiarism.

Next, Galileo, often presented as the craftsman of the mathematisation of nature and one of the founders of the modern scientific method, which postulates that only experimentation can serve as an arbiter of conjectures. Yet with regard to the fall of bodies, no one has been able to reproduce his results, at least not with the precision Galileo advances. It therefore seems that Galileo, seduced and convinced by the correctness of his mechanical theory, transformed a thought experiment into a physical experiment by twisting the figures (21). This is called data fabrication.

Finally, Mendel, the Czech monk who is considered to be the inventor of modern genetics. By crossing peas and observing the frequency of certain hereditary traits, he established the law of gene transmission, which is still valid today (22). The problem is that his results are statistically too perfect to be true. In today’s terms, this is called data tampering.

## Clearly, fraud is nothing new. But is it on the increase these days?

An article by Fang *et al.* published in 2012 is revealing (23). Its analysis of more than 2000 articles in the medical and life sciences fields, indexed in PubMed as retracted, revealed that three quarters were withdrawn for scientific misconduct – i.e. for fraud – and only one quarter for error. A 2009 survey by Professor Daniele Fanelli found that nearly 2% of medical researchers admitted to fabricating, falsifying, or altering data or results at least once, and some 15% said they had become aware of fraud committed by their colleagues (24). Those figures suggest that we are more inclined to call out colleagues than to own up to our own misdeeds.

The advent of digital technology has unquestionably facilitated scientific fraud… but digital technology has also made it easier to root out fraudsters. This is how Adam Marcus, editor of Gastroenterology and Fibroscopy, and journalist Arthur Carter co-founded Retraction Watch in 2011, a particularly valuable site whose function is to track and list retracted articles in journals (https://retractionwatch.com/). In this regard, a distinction should be made between an article that is rejected by an editorial committee and which, consequently, is not published, and an article which has been published and which the editorial committee later decides to withdraw… sometimes at the request of the author who becomes aware of certain shortcomings or errors, but most often due to scientific misconduct.

## What are the principal ways in which fraud is committed?

We have a wide range of dubious scientific practices to choose from, everything from simple negligence to outright skulduggery. But several principal modalities can be identified:

- The fabrication, falsification, and/or modification of data or results.
- The embellishment of the data, which consists in keeping only the measurements that support the preconceived idea. This is a minor form of fraud, like the scientific bias that slips incognito into the study in progress, sometimes in good faith.
- More serious is the conflict of interest, which encourages favouring results that are favourable to funders.
- To these, we can also add plagiarism and the replication of a publication in another journal, which are both frequent examples of scientific misconduct.

## What can be the reasons or motivations for someone to commit fraud in the first place?

When it comes to fraud, conscious, and unconscious psychic processes are at work. Obscuration and the intimate conviction of being right inevitably lead to twisting reality’s neck. This is the case of Galileo, saved by the accuracy of his thought experiment. This is also probably the case with Raoult, although his assertions have been contradicted by indisputable facts. Dogged faith in the correctness of an idea when it is false sooner or later leads to conscious and organised fraud, in the hope of saving face. So, a fraud committed knowingly, and for which, we might say, one assumes full responsibility.

Moreover, the current system by which we evaluate researchers – who must publish quickly and frequently in order to obtain funding – accentuates the risk of fraud. Add to this the media pressure that demands results – or at the very least hope – in the face of difficult situations. A prime example? That hasty announcement of positive results for tocilizumab, which we touched on earlier. We can also mention the race for big data: millions of data are collected and crunched in a computer, under the illusion that indisputable revelations will emerge, rather than taking the time to meticulously analyse the facts. In a world where beating out the competition tends to overshadow knowledge, abuses become inevitable.

**What are the consequences of a proven fraud**, a fraud that requires an article to be retracted?

To begin with, when fraud is revealed, it inflicts a deep trauma.

First, because they are vilified and censured – trauma for the fraudster. Then, more broadly, trauma for the fraudster’s immediate circle – friends and colleagues who are seized by feelings of anger, disbelief, and sometimes guilt. Relevant questions are raised – about the future image of the laboratory or clinical service; about the articles that the fraudster has already published; about the legitimacy of funding that was allocated for research that has now been exposed as fraudulent.

The situation for the whistle-blower is no less problematic. It is a daunting step to denounce a fraud. Anyone who sounds the alarm begins a journey that is fraught with pitfalls: trying to reproduce the experiment; facing colleagues who published with the fraudster; facing the experts who are charged with verifying the merits of the complaint; and confronting the editors and publishers of scientific journals, so that the fraudulent work is retracted with complete transparency.

The most destabilising moment for everyone is when the retraction becomes a publicly known fact. At that point, it will be commented on freely and widely in the mainstream media, and on social networks. Ultimately, the image of science and scientists becomes suspect in the eyes of the greatest number, inducing regrettable suspicions of conspiratorial behaviour.

## Let us sum up

What the general public is discovering, particularly through the controversies surrounding the Covid-19 pandemic, is quite simply the normal process of how we acquire knowledge. We do this through trial and error, controversy, and sometimes even mystification. This is what makes science science. It is also what makes scientific knowledge refutable, which is not the case with pseudoscience or religious beliefs.

Oscar Wilde once memorably confessed, ‘I can resist everything except temptation’ (25). I dare say we are all very much like Oscar Wilde. So, with Oscar’s words in mind, I would like to conclude with a word of advice for up-and-coming surgeons.

If you have an idea – let us say an excellent idea, an idea you are convinced is right – please hasten to make it known by means that are easily accessible: a case report, a letter to the editor, or a preliminary report in a second-tier journal. Now, I will grant you: that is not a very glorious way to go about it. But here is the upside: it will give other teams a chance to examine the validity of your idea. I can think of no better way to save yourselves from temptation – the ever-present temptation to commit fraud.

## Declaration of interest

The author declares that there is no conflict of interest that could be perceived as prejudicing the impartiality of the work reported.

## Funding

This work did not receive any specific grant from any funding agency in the public, commercial, or not-for-profit sector.
